# Understanding the Relationship Between Ecological Momentary Assessment Methods, Sensed Behavior, and Responsiveness: Cross-Study Analysis

**DOI:** 10.2196/57018

**Published:** 2025-04-10

**Authors:** Diane Cook, Aiden Walker, Bryan Minor, Catherine Luna, Sarah Tomaszewski Farias, Lisa Wiese, Raven Weaver, Maureen Schmitter-Edgecombe

**Affiliations:** 1 Department of Psychology, College of Arts and Sciences, Washington State University, 3160 Folsom Blvd, Sacramento, WA, 95816, United States, 1 5093354985; 2 Department of Neurology, UC Davis Medical Center, University of California at Davis, Davis, CA, United States; 3 Christine E Lynn College of Nursing, Florida Atlantic University, Boca Raton, FL, United States

**Keywords:** ecological momentary assessment, smart home, smartwatch, cognitive assessment, well-being, monitoring, monitoring behavior, machine learning, artificial intelligence, app, wearables, sensor, effectiveness, accuracy

## Abstract

**Background:**

Ecological momentary assessment (EMA) offers an effective method to collect frequent, real-time data on an individual’s well-being. However, challenges exist in response consistency, completeness, and accuracy.

**Objective:**

This study examines EMA response patterns and their relationship with sensed behavior for data collected from diverse studies. We hypothesize that EMA response rate (RR) will vary with prompt time of day, number of questions, and behavior context. In addition, we postulate that response quality will decrease over the study duration and that relationships will exist between EMA responses, participant demographics, behavior context, and study purpose.

**Methods:**

Data from 454 participants in 9 clinical studies were analyzed, comprising 146,753 EMA mobile prompts over study durations ranging from 2 weeks to 16 months. Concurrently, sensor data were collected using smartwatch or smart home sensors. Digital markers, such as activity level, time spent at home, and proximity to activity transitions (change points), were extracted to provide context for the EMA responses. All studies used the same data collection software and EMA interface but varied in participant groups, study length, and the number of EMA questions and tasks. We analyzed RR, completeness, quality, alignment with sensor-observed behavior, impact of study design, and ability to model the series of responses.

**Results:**

The average RR was 79.95%. Of those prompts that received a response, the proportion of fully completed response and task sessions was 88.37%. Participants were most responsive in the evening (82.31%) and on weekdays (80.43%), although results varied by study demographics. While overall RRs were similar for weekday and weekend prompts, older adults were more responsive during the week (an increase of 0.27), whereas younger adults responded less during the week (a decrease of 3.25). RR was negatively correlated with the number of EMA questions (*r*=−0.433, *P*<.001). Additional correlations were observed between RR and sensor-detected activity level (*r*=0.045, *P*<.001), time spent at home (*r*=0.174, *P*<.001), and proximity to change points (*r*=0.124, *P*<.001). Response quality showed a decline over time, with careless responses increasing by 0.022 (*P*<.001) and response variance decreasing by 0.363 (*P*<.001). The within-study dynamic time warping distance between response sequences averaged 14.141 (SD 11.957), compared with the 33.246 (SD 4.971) between-study average distance. ARIMA (Autoregressive Integrated Moving Average) models fit the aggregated time series with high log-likelihood values, indicating strong model fit with low complexity.

**Conclusions:**

EMA response patterns are significantly influenced by participant demographics and study parameters. Tailoring EMA prompt strategies to specific participant characteristics can improve RRs and quality. Findings from this analysis suggest that timing EMA prompts close to detected activity transitions and minimizing the duration of EMA interactions may improve RR. Similarly, strategies such as gamification may be introduced to maintain participant engagement and retain response variance.

## Introduction

### Background

Ecological momentary assessment (EMA), also known as experience sampling or in situ (situated) self-reporting, is a powerful method for capturing real-time insights into an individual’s health and well-being. Leveraging the convenience and ubiquity of mobile devices, EMA has been particularly effective in longitudinally monitoring conditions such as depression and mental well-being [1,2], mobility [3], physical activity [4], and fatigue [5].

The strength of EMA lies in its ability to minimize recall bias [6,7] and provide more fine-grained longitudinal data compared with traditional observation methods or retrospective reporting [8,9]. Participants can answer questions without traveling to a research lab, reducing a burden that is already great for individuals with Alzheimer disease and related dementias. Data collected through EMA reveal both within-day and between-day fluctuations in health and well-being [10], laying a foundation for timely, in-the-moment interventions. Furthermore, these responses provide a rich source of patient-reported outcome measures. When combined with objective, sensor-based data representing digital phenotypes, EMA enables the comparison of digital behavior markers with self-reported data.

Despite these advantages, EMA implementation faces challenges, especially in the variability, completeness, and accuracy of participant responses to prompts. Factors such as distraction, self-awareness, boredom, time of day, and interruption burden [11] can impact participant responses. Addressing these issues is essential for maintaining the integrity of research findings. Furthermore, the design of notification strategies may dramatically impact response compliance and quality [12,13].

In this paper, we conduct an examination of EMA response completeness and quality across a spectrum of settings. By leveraging data from multiple studies, we enhance the robustness and generalizability of our conclusions. This broad approach also allows us to consider variables previously unexplored in EMA research. By correlating EMA responses with sensor-derived biomarkers, we will better understand the contextual factors influencing participant engagement with EMA prompts. The findings from this study will inform refinements in EMA methodology and guide future research to improve the reliability of EMA-derived data and the effectiveness of just-in-time interventions.

### Previous Work

In-the-moment sampling builds a foundation for designing effective just-in-time interventions [14]. McDevitt-Murphy et al [15] used such a technique to combat anxiety by prompting individuals to write about their worries and initiate physical activity. Similarly, Mair et al [16] found such interventions improved physical activity in older adults, while Perski et al [17] constructed a technology-driven in-the-moment intervention to reduce substance abuse and promote healthy behaviors. Brenner and Ben-Zeen [18] found that letting individuals see the difference between their predicted future EMA reports of mood and actual EMA responses improved the effectiveness of their intervention.

EMA benefits must be weighed with the cost of an increased interruption load. Frequent requests for self-report often lower responsiveness [13]. Many factors have been considered that impact responsiveness. These include the impact of EMA prompts while a person is performing physical activities [19,20] or activities that require concentration [21]. Jeong et al [22] and Rintala et al [23] studied the relationship between a person’s location (eg, at home vs away from home) and responsiveness, while Pizza et al [24] and Ziesemer et al [25] found that engagement in social activity was an important consideration. Several studies consider whether prompt time of day influences likelihood of response [12,26-30]. Aminikhanghahi et al [31] found that prompting during transitions between activities improved participant responsiveness. Recently, investigators observed a relationship between the complexity of an EMA interaction and responsiveness [32]. They responded by shortening the questions [33], simplifying the interface [21], and making use of voice input [34].

Researchers also observed that the quality of responses decreases over time [35], becoming bistable or skewed [36], more habitual [37], and less variable [38-40]. Such trends threaten the reliability of research results. For example, Verbeij et al [41] observed decreased convergent validity between reported and objective social media use over 3 weeks of reporting [41].

These findings allow researchers to better design EMA-based studies and control for such factors in their analysis. One difficulty, though, is that some of the findings are contradictory. While Jeong et al [22], McMillan et al [27] and Ziesemer et al [25] reported higher response rates (RRs) when a person was alone, Pizza et al [24] observed no significant impact of social setting and Ponnada et al [26] reported a higher RR when a person was with family or friends. Regarding time of day, some studies found that participants responded more often during afternoons and evenings [26,28,42], while others [43,44] observed more responses early in the day. Khanshan et al [19] found RRs to be higher while participants were active, while Boukhechba et al [29] found higher activity was correlated with missing more responses. These discrepancies may indicate the influence of other population and study parameters. We address the need to consider these factors by performing a multistudy analysis.

Furthermore, Wrzus and Neubauer [45] emphasized the need to employ sensors in the understanding of the interplay between participant behavior, EMA responsiveness, and response quality. Stach et al [46] observe that mobile notification responsiveness is influenced not only by the app delivering the notification but also by user demographics. In our analysis, we leveraged continuously collected sensor data to examine the relationship between EMA responses (as patient-reported outcome measures), digital markers extracted from sensor data (digital phenotyping), and demographics across multiple studies. This comprehensive approach enabled us to uncover patterns and develop recommendations for optimizing EMA methodologies.

### Study Goal and Hypotheses

In this study, we performed statistical analysis of EMA responses and sensor data from 9 clinical studies. The overall goal was to provide insights into EMA response patterns that can be used to design and improve future in-the-moment monitoring and intervention studies. We analyzed data from 454 participants to validate the following hypotheses:

First, EMA RR will vary with prompt time of day, number of questions, and sensor-observed activity.

Second, EMA response quality will decrease as a function of the number of days that have elapsed in the study.

Third, both RR and response quality will be impacted by a participant’s behavior context, as modeled by markers extracted from mobile data.

Fourth, differences in EMA RRs and response patterns will exist between studies, influenced by participant demographics (ie, participant age) and study purpose (ie, cognitive intervention vs observational).

## Methods

### EMA Studies

Daily EMA responses were collected between October 2015 and December 2023 for a total of 454 participants across 9 distinct studies. Reporting of the EMA steps is consistent with the STROBE CREMAS (Strengthening the Reporting of Observational Studies in Epidemiology Checklist for Reporting Ecological Momentary Assessment Studies) guidelines [47]. In each study, participants received prompts to answer EMA questions using an in-house app running on a smartwatch or tablet. Participants were trained on how to interact with the app by a research assistant and completed practice prompts and tasks before data collection commenced.

The EMA prompts were randomly distributed within predefined time blocks throughout the day. We adjusted these time blocks when necessary to fit participant schedules. When a prompt was issued, an audio tone was played to alert the participant. The participant would then read the question and select their response from options on a mobile screen. If there was no response within 5 minutes, the prompt was reissued, up to a maximum of 5 attempts (2 attempts for dyad study). If the participant still did not respond, we recorded this as a nonresponse and did not issue further prompts until the next designated time block. Parameters for the studies are summarized in Table 1. In total, these comprise 145,853 EMA prompts with 115,283 sets of responses.

**Table 1.** Ecological momentary assessment studies that were included in the analysis.

Study	Population	Sample^a^ (n)	Prompts (n)	Questions and tasks: number and topic	RR^b^ (%)	Duration
Rural (mc)	Aged 50+ y, cognitive health spectrum, Florida	33	9200	8 (social contact, physical activity, mental activity, environment, and motivation), *n*-back^c^	77.79	2 weeks, day
Function (func)	Aged 50+ y, cognitive health spectrum, Pacific Northwest	26	17,923	11 (social contact, physical activity, mental activity, environment, and activity type), *n*-back, audio^d^	73.85	5 weeks, spaced over 16 months, day+night
Note-book (mn)	Aged 50+ y, cognitive health spectrum, Pacific Northwest	45	17,369	15 (memory strategy, confidence, activity type, environment, and emotion analysis), [*n*-back, audio^d^]^e^	79.76	2 weeks, 6 months apart, day
Brain Booster (bb)	Aged 65+ y, subjective cognitive decline, California	184	3207	12 (environment, concentration, and emotion analysis), *n*-back 1x/day	54.35	2 weeks, 6 months apart, day
Compen-satory (cs)	Aged 50+ y, primarily cognitively healthy, Pacific Northwest	89	45,423	11 (activity type, environment, memory strategy, and location), *n*-back, audio^f^	85.09	2 weeks, day
Smart-Home (sh)	Aged 55+ y, cognitively healthy, Pacific Northwest	43	32,065	7 (social contact, physical activity, mental activity, and activity type), *n*-back	78.86	2 weeks, 1 month apart
Dyad (dyad)	Aged 55+ y, cognitively healthy, West Virginia and Virginia	10	5173	8 (dyad interactions and emotion analysis), audio^d^	91.51	2 weeks, day+ night
Gsur1 (gsur1)	Students, cognitively healthy, Pacific Northwest	6	3727	12 (social contact, physical activity, mental activity, environment, and activity type), *n*-back	84.52	2 weeks, day
Gsur2 (gsur2)	Students, cognitively healthy, Pacific Northwest	18	12,666	15 (social contact, physical activity, mental activity, environment, activity type, location, and emotion analysis), *n*-back, audio^d^	59.01	2 weeks, day

a Number of participants enrolled in the study (through December 2023).

b RR: response rate.

c *n-*back is a 45-second 1-back shape task.

d Audio prompt to describe their day.

e Additional tasks introduced late in the study (31.2% of mn participants).

f Audio prompt to describe what tasks were assisted by memory strategies that day.

Well-being has been defined multiple ways in the literature [48-51]. Many studies rely on 1-time questionnaires, like the Satisfaction With Life Scale [52], to measure well-being at a single time point. However, well-being is dynamic, fluctuating across different times and contexts [53]. EMA allows us to capture individuals’ daily behaviors within their natural environments alongside real-time self-reports on well-being. In this analysis, we focus on three dimensions of well-being: mental sharpness, physical fatigue, and stress.

**Figure 1.**
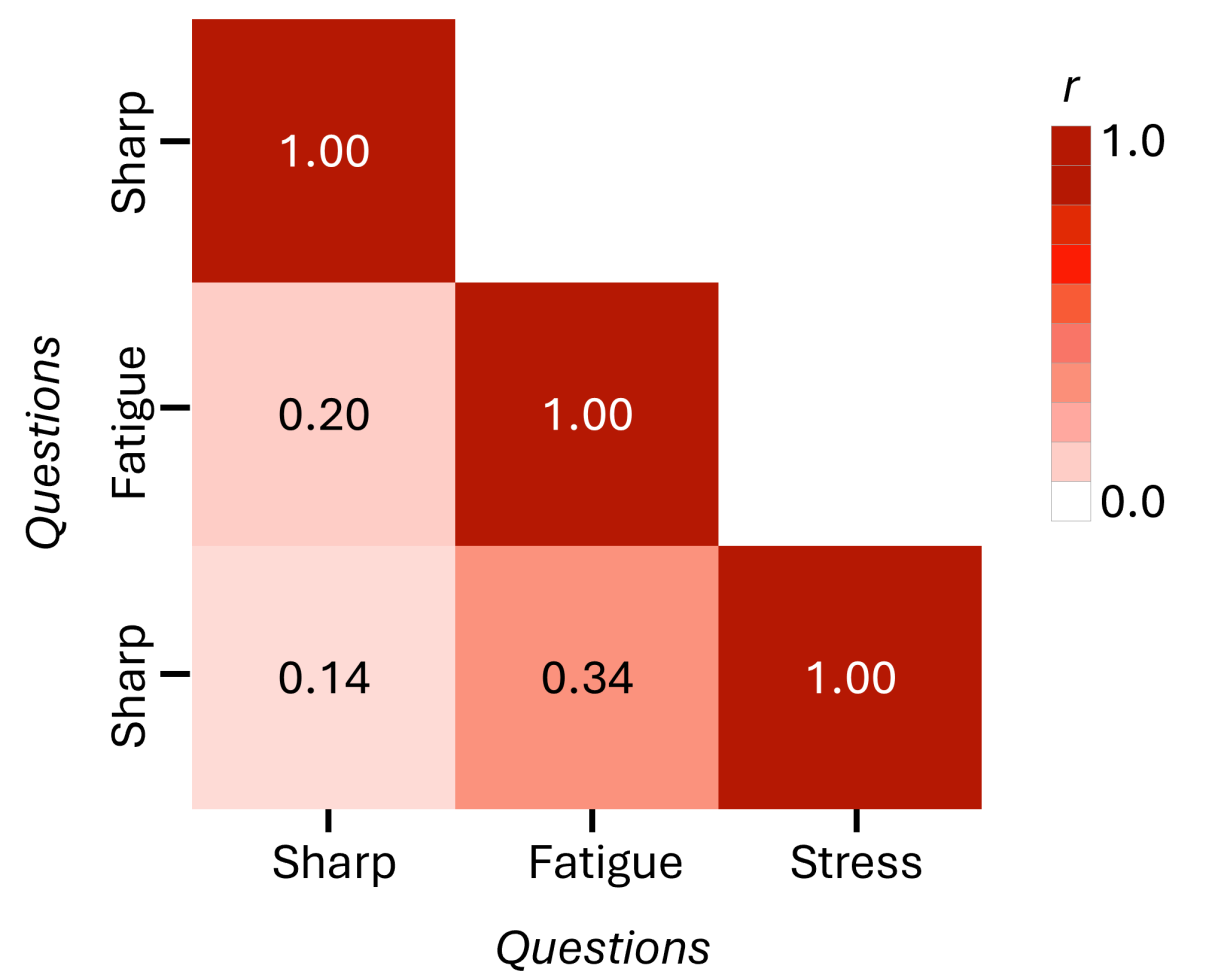
Correlation between participant responses to the three question types.

Each study included subjective questions that examined (1) $Q_{sharp}$ = the person’s mental sharpness, (2) $Q_{fatigue}$ = the person’s physical fatigue, and (3) $Q_{stress}$ = the person’s level of stress, all self-reported and relevant to the point in time (eg, immediately and up to the previous 2 h) when they received the query. The Likert scales varied between studies, so we normalized the values to a 1‐5 range. Where needed, we inverted responses for negative questions so that a higher-valued response is more positive in each case (eg, $Q_{sharp}=5$ indicates feeling greater amounts of mental sharpness; $Q_{fatigue}=5$ indicates feeling less physically fatigued; $Q_{stress}=5$ indicates feeling less stressed. Multimedia Appendix 1 plots the relative frequency of each response to the 3 question categories. The studies are ordered primarily by the cognitive health status of participants and secondarily by age. As the plots demonstrate, most of the responses are high but do vary among studies. Notably, the similarity in response between stress and fatigue is higher than other question pairs. This finding is consistent with the correlation that exists between responses to the 3 questions, shown in Figure 1.

### Ethical Considerations

These studies were reviewed and approved by the institutional review boards (IRB protocol 14460) at Washington State University and the University of California at Davis (No. 19784). To participate in any of the studies, participants needed to understand English and sign an informed consent; each person received compensation between US $20 and US $125 for their participation. All data were anonymized before performing analyses.

### Statistical Analysis

#### RR and Quality

We conducted statistical analyses on the data to examine RR, completeness, and quality for each participant and study. Our app records each time a prompt is delivered to the participant. From these records, we calculate RR as the ratio of occurrences when a participant answered at least 1 question to the total number of prompts. The analysis also considers the prompt time of day (morning before 12 PM, afternoon between 12 PM and 5 PM, and evening after 5 PM), day of week, study duration, and number of questions per prompt. Researchers suggest that user demographics may impact preference for EMA delivery strategies [21]. We examine this factor by considering differences among the studies and between the set of studies involving older adults (aged 50 y or older) and those involving younger adults.

Response completeness reflects the percentage of questions that were answered for the occasions when the participant responded. We excluded nonquestion tasks, such as daily *n*-back shape tests (all but dyad) and audio descriptions of the day’s activities (func, mn, cs, dyad, gsur1, and gsur2). Rather than impute missing values, analysis of EMA-reported values only considers completed responses.

Response quality measures habituation that may occur over the duration of the study, resulting in less response variance and state awareness. As reported elsewhere [54-56], we quantified careless responses as the percent responses that fall near (within 0.2) each participant’s mode response and also calculated response variance. Changes in response quality may occur over the course of the study as a motivation to provide high-quality responses wanes. Thus, we computed the change in variation and careless responses between the first and second half of the study for each participant.

To gather sensor-derived behavior context, we collected continuous sensor data from Apple watches for 95 participants in the bb study and all participants from the dyad, func, cs, mn, mc, sh, gsur1, and gsur2 studies. In the ihs study, we collected data from ambient sensors installed in participant homes. The smartwatches collected acceleration and rotation readings at 10 Hz and recorded location (latitude, longitude, and altitude) every 5 minutes, or more frequently when movement exceeded a baseline. Data were stored on the watch, encrypted, and downloaded to a password-protected server when the watches were returned. From the raw location data, we defined the participant’s home as the location visited most often between 2 AM and 9 AM. We then calculated the distance and bearing from home for each location. Behavior context features were defined as “activity level” (total acceleration) and time “at home” (fraction of time spent at home), calculated for 30 minutes ending at the time the participant provides an EMA response. The data collection app was developed in-house using Swift. Collecting data at these rates ensured 1 full day of data on a single charge.

For studies collecting data only during the day (Table 1), participants wore the watch on their nondominant arm during the day and charged it at night. In studies collecting both day and night data, participants were given 2 watches—one worn during the day and charged at night, and the other worn at night and charged during the day. In the smart home study, EMA responses were reported using a tablet; in all other studies, they were reported via the smartwatch.

In the smart home study, 2‐4 sensors were placed in each room. These were either passive infrared motion sensors with an ambient light sensor or magnetic door sensors with an ambient temperature sensor. Motion sensors detected movement, providing location data within the home. Activity level was estimated by counting motion sensor readings. If an external door was opened and closed, followed by at least 5 minutes of no motion sensor activity, the participant was designated as out of home. The “at home” context quantified the fraction of time spent at home in the 30 minutes before an EMA response.

Another context feature was proximity to a change point. A change point occurs when there is a shift in the underlying process of a time series, such as transitioning from one activity to another. In an earlier work by Aminikhanghahi et al [57], we designed an algorithm to detect these points from continuous sensor data and found that interacting with participants during these times improved RRs and task success. We used the same method here to determine proximity to a change point.

We calculated correlations between the 3 context features (activity level, at home, and change point) and response compliance. Here, a lack of response to a particular prompt is assigned a “compliance” value of 0.0, a complete response is assigned a value of 1.0, and a partial response (some questions answered, some not) is assigned in the range 0.0‐1.0, corresponding to the relative number of questions that were answered.

#### Study Differences

In this analysis, we selected 9 studies as the basis for comparing EMA delivery strategies and responses. The selection was based on the consistency of the technical data collection tools and hardware, which minimized the influence of these factors on the results. A single software interface was used to deliver EMA prompts across all studies, and the same app collected sensor data in all smartwatch studies, following a protocol similar to that used in the smart home study. Context features were extracted uniformly from the sensor data across datasets.

Participants in all studies were instructed to perform their normal daily routines without changing their behavior to accommodate the data collection. This consistency allowed us to focus on the differences that existed between the settings. Specifically, the participant groups differed across studies in terms of location (4 regions within the United States), age, and cognitive health, supporting generalization of the findings to other EMA studies. The studies also differed in length and number of EMA questions and tasks. These differences represent the focus of our analysis.

We examined differences in EMA response dynamics across the 9 studies. Our approach is twofold; we report results for each study individually and collectively and analyze differences in EMA responses within and between these studies. This technique will allow us to examine the extent to which EMA response patterns differ between studies, using within-study changes as a comparison baseline. We use dynamic time warping (DTW) as a similarity measure because the technique measures similarity between time series that differ in length as well as values. In the case of the $Q_{sharp}$ question, we also visually compare responses over time for studies that focus on cognitive health support for older adults (bb and mn) with observational studies involving cognitively healthy adults (dyad, gsur1, and gsur2).

To deepen the analysis, we fit ARIMA (Autoregressive Integrated Moving Average) models to the aggregated EMA responses for each question type. The mean response was computed across participants for the first 48 responses, producing a single representative time series. The resulting model parameters indicated how well the shape of responses over time could be modeled.

## Results

### RR Findings

RR across all participants was 79.95%. This is within 0.76% of the 79.19% average rate Wrzus and Neubauer [45] reported in a survey of 417 EMA studies. Among the prompts that received participant responses, the response completion rate was 88.37%.

**Figure 2.**
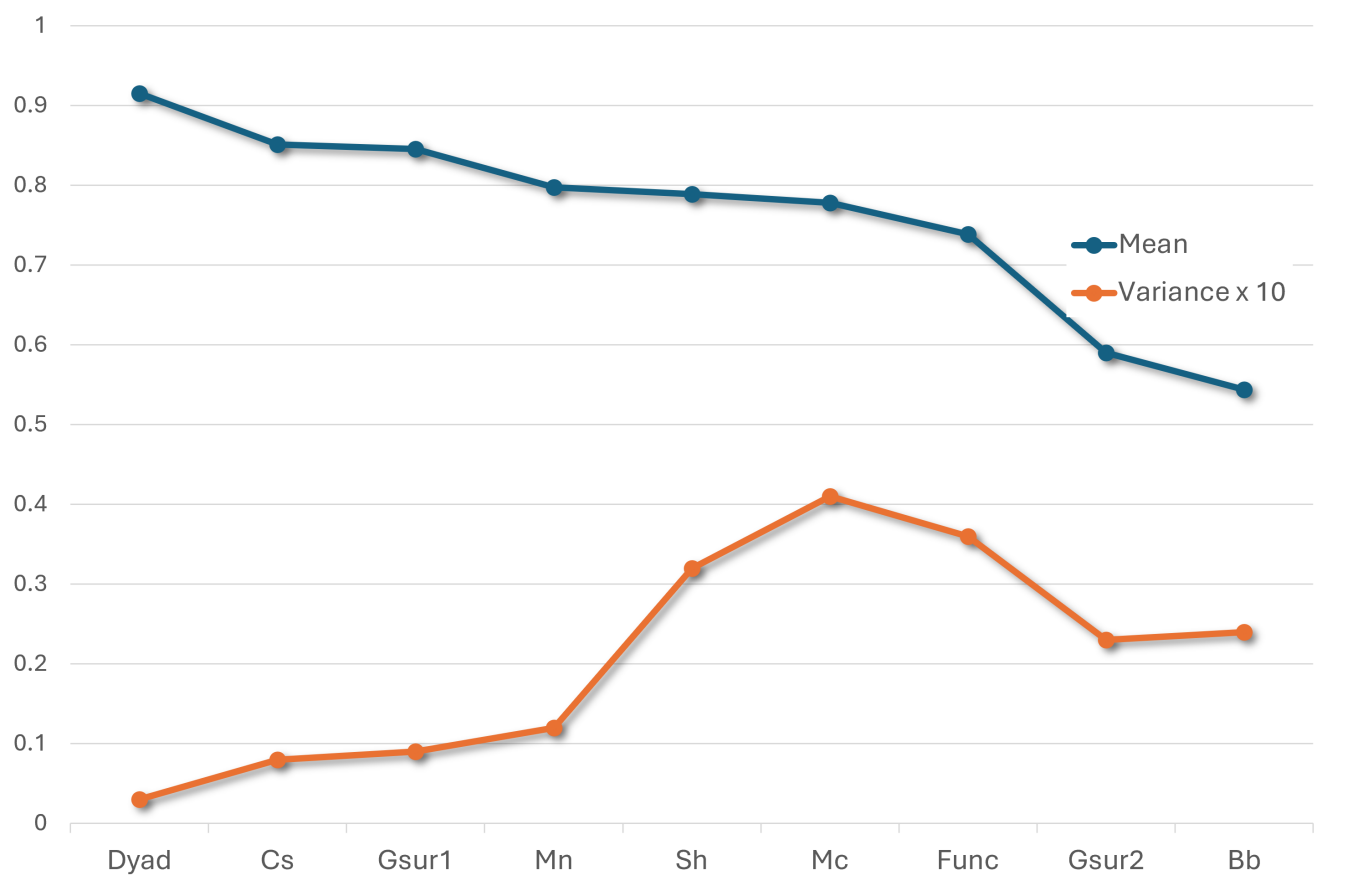
Response rate mean and variance. Rates are shown in sorted order; variance is multiplied by 10 to align the plots.

Figure 2 plots RR for the studies, ordered from largest to smallest. Response variance is quite different among the studies. Notably, response variance increased as the mean RR decreased. Table 2 shows the overall most popular time of day for responding to EMA prompts. However, these results vary based on participant age. Only considering studies with older adults (age 50 y or older), participants were more responsive in the evening than morning (by 3.95) or afternoon (by 3.58). In contrast, younger adult RR remained fairly uniform throughout the day, with afternoon showing the highest rate. Weekday and weekend prompts yielded very similar RR overall (a difference of 0.04). However, this varied by study and age group. Specifically, while weekend response was higher by 3.25 for younger adults, the rate decreased for older adults by 0.27. These findings highlight the role that participant demographics play in understanding EMA responsiveness; prompt time alone may not be a strong indicator of responsiveness.

**Table 2.** Response rate (%) based on prompt time of day and day of week.

Time		Overall response rate (%)	Response rate among older adults (%)	Response rate among younger adults (%)
**Time of day**				
	Morning	79.02	80.60	64.86
	Afternoon	79.08	80.97	64.97
	Evening	82.31	84.55	64.45
**Day of week**				
	Weekday	80.43	82.46	63.93
	Weekend	80.39	82.19	67.18

**Figure 3.**
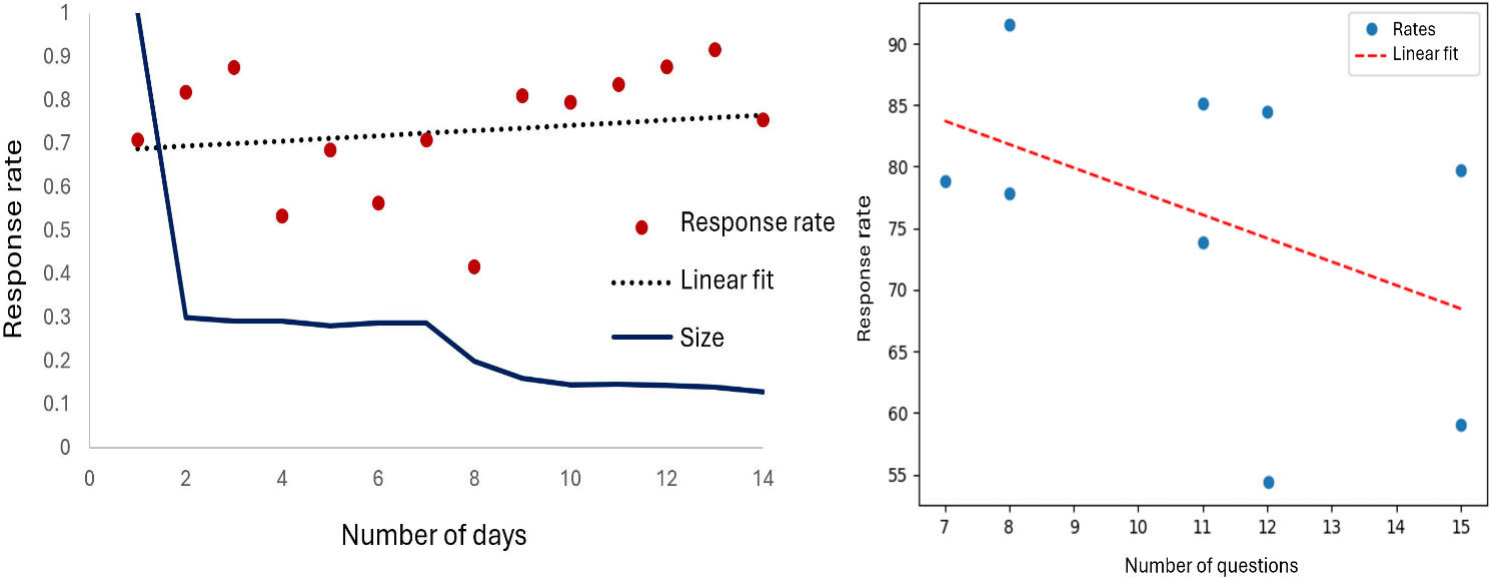
Left: response rate based on number of study days and relative sample size at that time point (fraction of original sample). Right: response rate as a function of the number of questions asked at the EMA prompt.

Figure 3 (left) plots EMA RR as a function of the number of days that elapsed since the beginning of the study for each participant. The linear fit line has a positive slope of 0.078, indicating that the duration does not negatively impact RR. At the same time, the fraction of participants still collecting data is much smaller at the end of this time span. A nonsignificant small positive correlation exists between the study duration and RR (*r*=0.100*, P*=.29). This may be due to the EMA prompts becoming more integrated into the participant’s routine and their gaining familiarity with the questions and methods of responding.

Figure 3 (right) shows RR as a function of the number of questions asked at the EMA prompt, together with the fitted line. Correlation between the number of questions asked during an EMA prompt and RR is moderate and negative (*r*=−0.433, *P*<.001). The line fitting this trend has a slope of −1.90. This finding is consistent with previous work indicating that individuals are less likely to respond when each EMA prompt requires a large time interruption and corresponding task burden [37].

### Response Quality

Table 3 summarizes participant responses and response quality. The average response value across all studies is relatively high, as is the percentage of responses near the mode. There was a noticeable shift toward response uniformity over time, evidenced by a significant increase in carelessness and decrease in variance. Results from individual studies are shown in Multimedia Appendix 2. Although the earlier analysis showed that RR did not decline over the study, the increase in careless responses and decrease in variance indicates a possible increase in habitual responses to EMA questions [37] as participants got bored or felt time pressure to respond to EMA notifications. Some studies inserted a break of 1+ months during data collection. For these studies, we calculated changes within the period before the break and the period after the break. The trends remain the same: carelessness increased and variance decreased over time. These changes are smaller, possibly due to the decreased time (typically 7 d) being examined.

**Table 3.** Summary of response averages and quality.

Metric	Value
$Q_{sharp}$ average response (variance)	3.524 (1.072)
$Q_{fatigue}$ average response (variance)	4.157 (0.758)
$Q_{stress}$ average response (variance)	4.432 (0.652)
Careless %	55.71
Interparticipant variance	0.611
Carelessness change	0.22 (*P*<.001)
Variance change	−0.363 (*P*<.001)
Prebreak carelessness change^a^	0.04 (*P*<.001)
Prebreak variance change^a^	−0.082 (*P*<.001)
Postbreak carelessness change^a^	0.09 (*P*<.001)
Postbreak variance change^a^	−0.329 (*P*<.001)

a func, mn, bb, and sh studies.

### Alignment With Sensor-Observed Behavior

Relationships between RRs and sensor-detected states were also examined. A correlation of *r*=0.045 (*P*<.001) exists between RR and activity level over the previous 30 minutes, *r*=0.174 (*P*<.001) between RR and time spent at home over the previous 30 minutes, and *r*=0.124 (*P*<.001) between RR and nearness to detected activity change points. Multimedia Appendix 3 summarizes the results for individual studies.

RR correlation with activity level is positive but small. This finding, considered together with the discrepancies observed in previous studies [19,29], may indicate that the nature of the person’s activity needs to be examined to understand the impact on prompt response. Correlations with at-home time and change points were larger, although still small. Individuals may not want to interrupt their activities, particularly in social situations, to answer lengthy EMA questions. In these contexts, they may ignore the prompt and wait until the next time block. As with our previous study examining RR and change points in smart homes [31], we observe that prompting near a change point improve responsiveness for all studies.

### EMA Differences Between Studies

Results from the previous analyses reveal that differences in EMA response compliance and quality may occur as a function of study and participant characteristics. Here, we delve further into EMA response differences between studies. First, we measure the distance (inverse similarity) between EMA time series using DTW. Across all studies and participants, the average within-study DTW distance was 14.141 (SD 11.957) overall (sharp 15.357, highest dyad=36.111, lowest bb=11.486; fatigue 15.014, highest dyad=36.489, lowest bb=10.299; stress 12.051; and highest dyad=33.511, lowest bb=7.069).

In contrast, the average between-study DTW distance was 33.246 (SD 4.971) overall (sharp 44.848, highest dyad/bb=81.448, lowest mc/bb=16.262; fatigue 29.365, highest mn/func=54.927, lowest mc/bb=12.434; stress 25.526, highest mn/func=52.05, lowest mc/bb=8.759). This represents a 135% distance increase over the within-study distances. The large increase indicates that while response variability exists between participants, an even larger difference exists between studies. Table 1 lists some of the parameters that may influence these differences; further investigation may identify more contributing factors.

**Figure 4.**
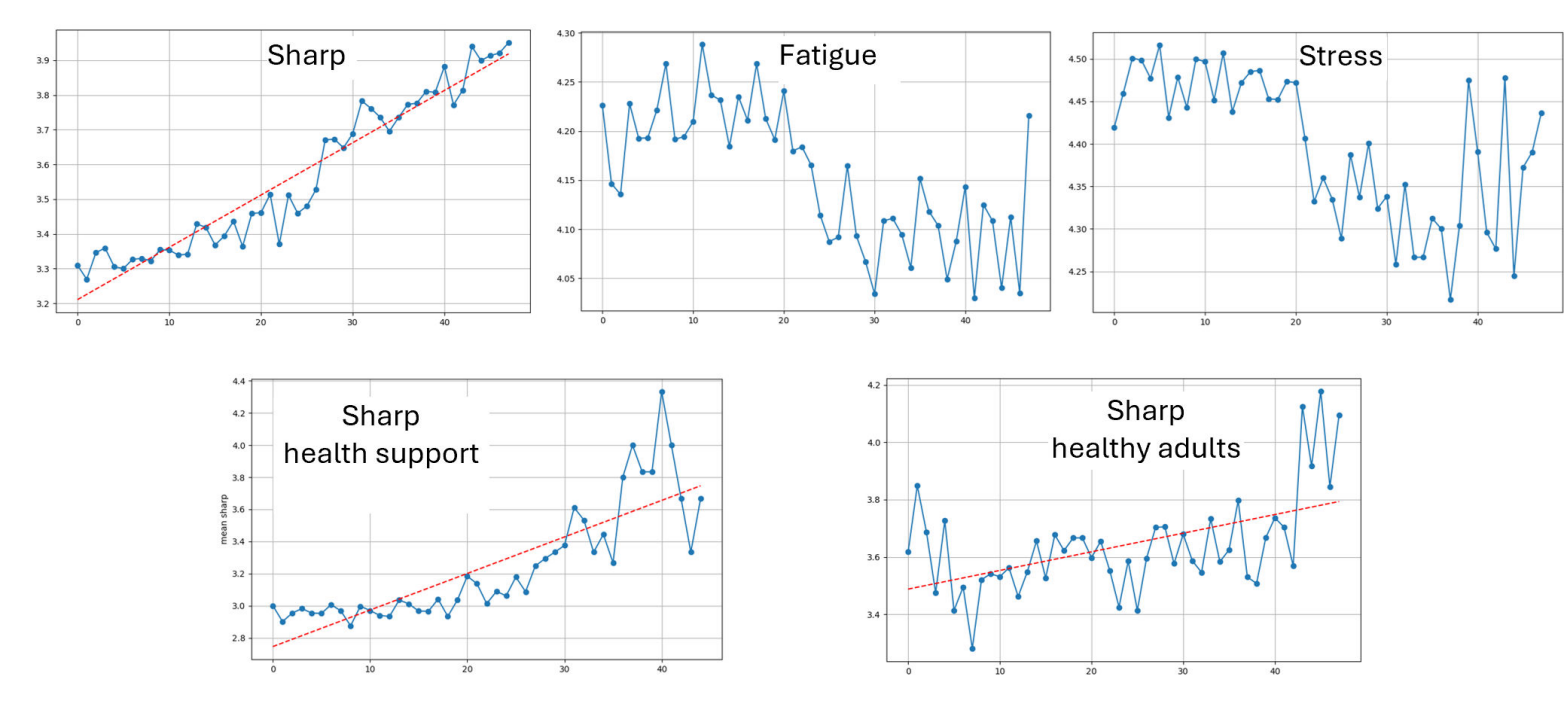
Aggregated ecological momentary assessment response sequences for (top left) $Q_{sharp}$, (top middle) $Q_{fatigue}$, and (top right) $Q_{stress}$. Aggregated ecological momentary assessment responses to the $Q_{sharp}$ question for (bottom left) cognitive health intervention studies and (bottom right) cognitively healthy adult studies. All $Q_{sharp}$ plots are accompanied by a linear fit line.

Plots of the aggregated EMA responses for the 3 question types are shown in Figure 4. The shapes of the aggregated time series are different between the 3 question categories. The $Q_{sharp}$ trend is consistently positive, while there are less noticeable trends for the other questions.

Because 2 of the studies (bb and mn) represent interventions, we examine differences in the response trends between these studies and the 2 observational studies with cognitively healthy older adults (dyad and cs). Aggregated over all 9 studies, the slope of the response fitted line is 0.018, with a y-intercept of 3.181. The fitted line for the intervention studies has a slope of 0.023 (an increase of 0.005) and y-intercept of 2.745 (a decrease of 0.436), while for observational studies with cognitively healthy older adults, the fitted line has a slope of 0.005 (a decrease of 0.013) and y-intercept of 3.449 (an increase of 0.614). Here, we observe that older adults receiving interventions start at a lower self-reported mentally sharp value but indicate that the sharpness increases over the study duration. In comparison, the cognitively healthy adults start at a higher sharp reported value and have a lesser amount of change in reported values during the study.

Table 4 presents the ARIMA parameters for the response categories sharp, fatigue, and stress. The log-likelihood values are high for all models, indicating a good fit. Similarly, the negative Akaike Information Criterion and Bayesian Information Criterion values suggest that the models strike a balance between fitting the data well and avoiding unnecessary complexity. The *P* values for the Moving Average of order 1 terms (*P*<.001 for all categories) indicate that errors at lag-1 are significant for predicting the next value in the time series. In addition, the Ljung-Box test results, with values greater than 0.05, suggest that the residuals are not significantly autocorrelated, meaning the models capture relevant patterns beyond mere autocorrelation.

**Table 4.** ARIMA (Autoregressive Integrated Moving Average) parameters for response categories.

Metric	Sharp	Fatigue	Stress
Log likelihood (higher is better)	74.19	72.39	64.09
AIC^a^ (lower is better)	−142.39	−140.79	−124.17
BIC^b^ (lower is better)	−136.84	−137.09	−120.47
MA(1)^c^	−0.63 (*P*<.001)	−0.67 (*P*<.001)	−0.61 (*P*<.001)
Ljung-Box test	0.85	0.21	0.07

a AIC: Akaike Information Criterion.

b BIC: Bayesian Information Criterion.

c MA(1): Moving Average of order 1.

## Discussion

### Principal Results

Early detection and intervention, crucial for reducing health care costs and enhancing quality of life, depend on timely reports of health status. This cross-study analysis highlights the pivotal role of EMA design in obtaining frequent, accurate EMA responses. In particular, we observed that participant demographics and study parameters markedly influence EMA response values, compliance, and quality. In particular, timing of EMA prompts varied in effectiveness depending on participant age. On the other hand, the negative impact of lengthy EMA interactions was consistent across all of the studies. Supporting eyes-free or hands-free interactions [21,34] may reduce interruption burden. Similarly, employing more frequent but shorter EMA sessions, or μEMA [26], may improve responsiveness. These findings support the first hypothesis and highlight the need to tailor EMA prompt strategies to specific participant and study characteristics.

A consistent observation was an increase in habitual, careless responses over the study duration, supporting the second hypothesis. In this study, 2087 of the 17,939 sessions (11.63%) were started but not completed, so researchers may consider strategies such as randomizing question or task order [58], gamifying the data collection process [8], or allowing selected questions to be skippable [59], as some may hold more interest. Additional factors, such as time pressure from concurrent activities or individual personality traits [55], may also influence responses. Ultimately, these considerations can be included in the study design.

Integrating sensor-based behavioral context is key to improving EMA studies. As hypothesized in the third hypothesis, our analysis revealed a significant relationship between sensor-derived biomarkers and EMA responsiveness. Analyzing these biomarkers could benefit personalized EMA design. In addition, timing EMA prompts close to detecting activity transitions may improve RR and quality.

Finally, our results showed that consistent with the fourth hypothesis, EMA response dynamics varied according to study parameters such as participant age and study type. We observed that response values increased more dramatically for intervention than observational studies, perhaps due to participants viewing their intervention involvement as beneficial, as noted in previous literature [60]. The response series were well fit by statistical models such as ARIMA. Such models provide a basis to compare EMA trajectories between groups and predict future response patterns.

### Limitations

This study had several limitations. Although we examined factors like study purpose and participant age, other variables, such as education, race or ethnicity, employment status, environment, and cognitive or physical health, were not examined. In addition, we did not consider the impact of breaks of 1 or more days in data collection. Restarting data collection after a break could affect EMA compliance and response quality.

Another limitation involves the duration of the studies. While some studies spanned 6 months or more, others lasted only 2 weeks. Extending the duration of all studies would enhance the generalizability of our time series findings. The sample sizes also varied considerably, with smaller young adult samples compared with older adult studies. In addition, slight differences in EMA question wording between studies, though normalized, may have influenced responses. Greater uniformity in future studies would provide more consistent data.

A further limitation was the lack of diversity in medical conditions. While we focused on individuals with varying levels of cognitive decline, other physical or psychiatric conditions were not represented. Including a wider range of medical conditions in follow-up studies would provide a more comprehensive understanding of EMA responses across different health conditions.

While the studies were consistent in their data collection hardware and software, technology influences results through factors like accessibility, battery life, ease of use, device familiarity, and extractable digital markers. Future studies can systematically consider alternative technologies.

Finally, this analysis concentrated on a subset of well-being factors. Since well-being encompasses broader emotional, social, and physical domains, future research can include additional dimensions for a more holistic analysis. In particular, a next step can be to evaluate the impact of rural versus urban residence as well as the quality of the build environment on EMA responses.

### Conclusions

This study offers insights into the factors that influence EMA responses and lays the groundwork for further exploration. Future studies should broaden the range of participant, study, and interaction characteristics to better inform EMA design strategies. In addition, expanding the range of sensor-based behavioral markers will enhance understanding of how changes in routine and behavior can enrich EMA data collection. Addressing these areas will help refine EMA methodologies, leading to more personalized and effective health monitoring and intervention technologies.

## Supplementary material

10.2196/57018 Multimedia Appendix 1 Bar charts illustrating the distribution of Likert responses for the sharp, fatigue, and stress ecological momentary assessment questions across studies. Higher scores are consistent with better well-being.

10.2196/57018 Multimedia Appendix 2 Left: percentage careless responses (within 0.2 of participant mode) and interparticipant variance (multiplied by 100 to normalize scale). Right: difference in percentage near the mode and variance between the first and second half of data collection for each participant.

10.2196/57018 Multimedia Appendix 3 Pearson correlation between ecological momentary assessment response and behavior context.

## Abbreviations

ARIMA: Autoregressive Integrated Moving Average
DTW: dynamic time warping
EMA: ecological momentary assessment
RR: response rate
STROBE CREMAS: Strengthening the Reporting of Observational Studies in Epidemiology Checklist for Reporting Ecological Momentary Assessment Studies
