# Supplementary figures and images for: Understanding the Relationship Between Ecological Momentary Assessment Methods, Sensed Behavior, and Responsiveness: Cross-Study Analysis
Source: JMIR Mhealth Uhealth. 2025 Apr 10;13:e57018. doi: 10.2196/57018 (PMC12005599; doi:10.2196/57018)

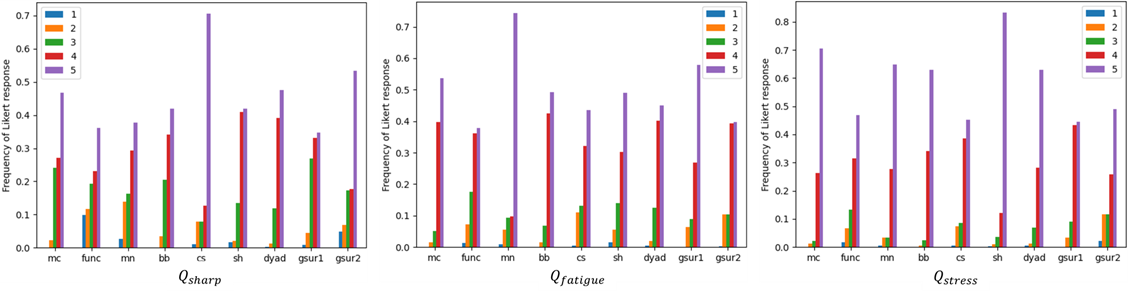

Supplement: Multimedia Appendix 1 [file mhealth-v13-e57018-s001.png]

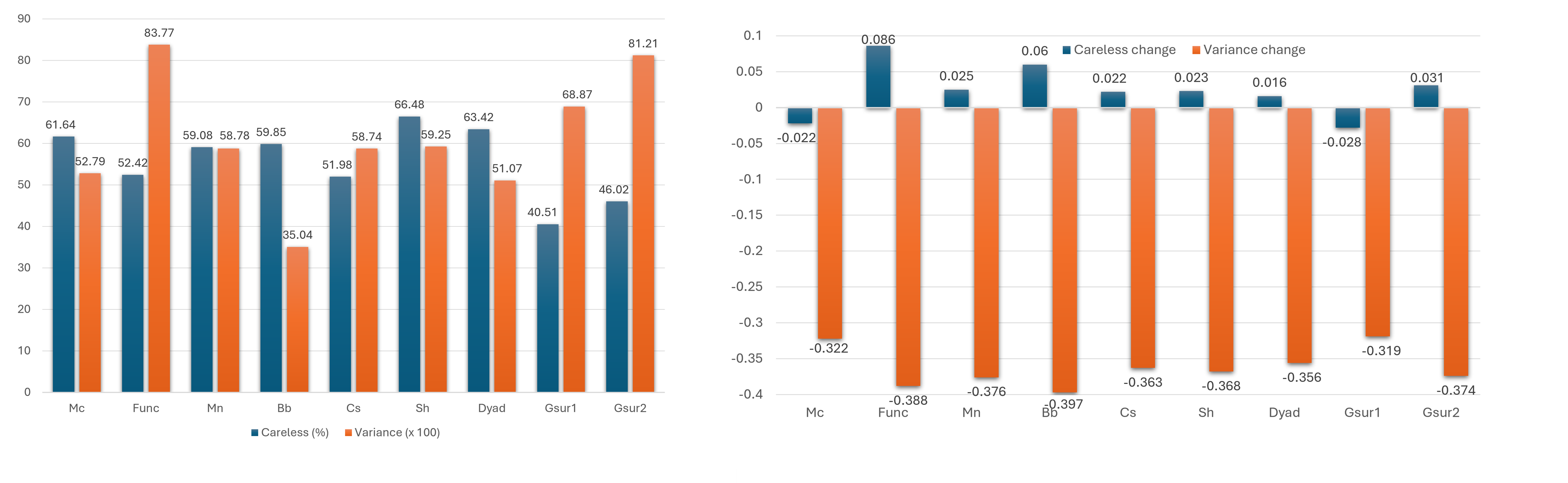

Supplement: Multimedia Appendix 2 [file mhealth-v13-e57018-s002.png]

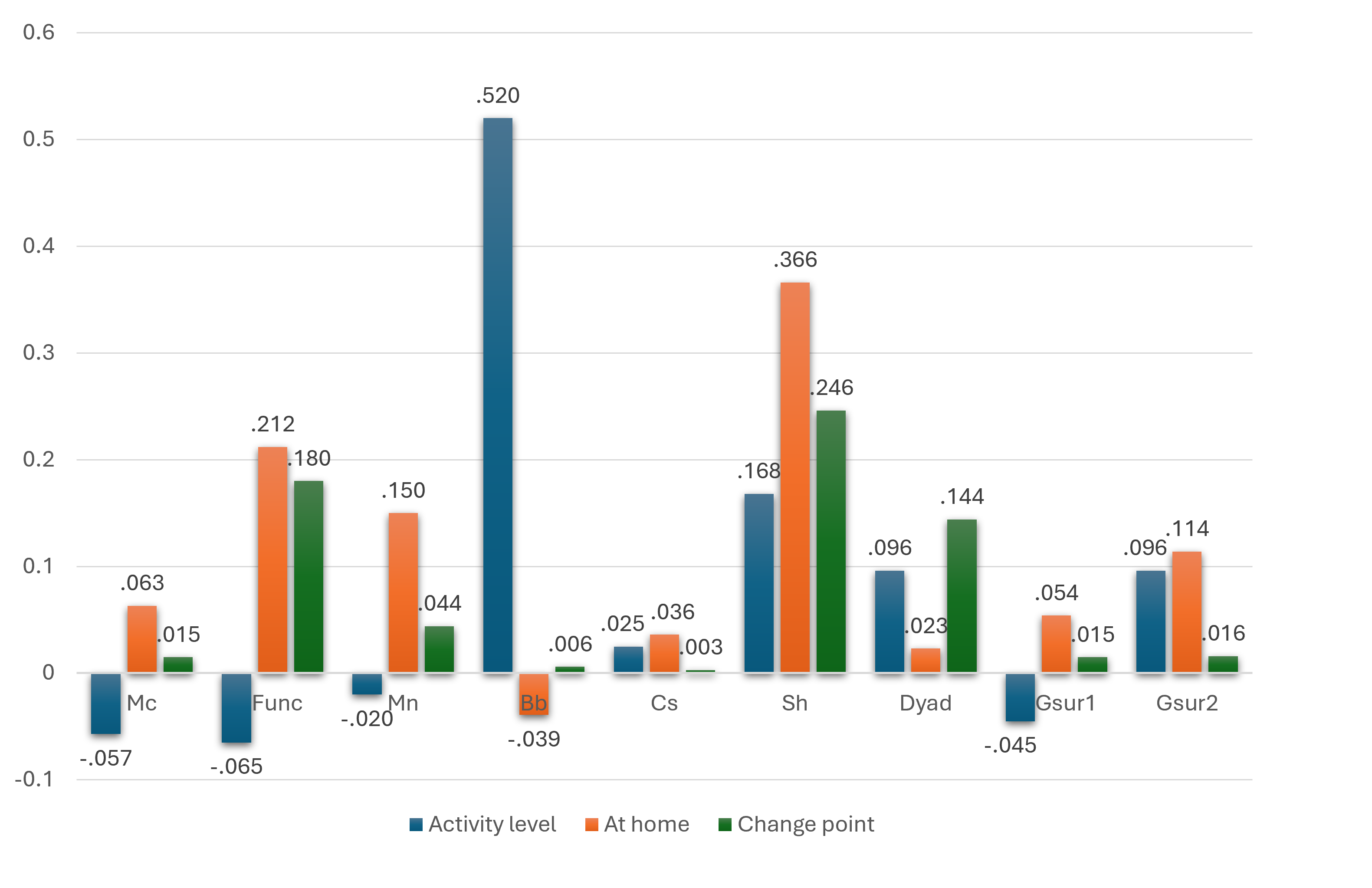

Supplement: Multimedia Appendix 3 [file mhealth-v13-e57018-s003.png]
